# Ganglioside GM3 Is Antiangiogenic in Malignant Brain Cancer

**DOI:** 10.1155/2010/961243

**Published:** 2010-06-20

**Authors:** Thomas N. Seyfried, Purna Mukherjee

**Affiliations:** Department of Biology, Boston College, Chestnut Hill, MA 02467, USA

## Abstract

Progression of malignant brain tumors is dependent upon vascularity and is associated with altered ganglioside composition and distribution. Evidence is reviewed showing that the simple monosialoganglioside, GM3, possesses powerful antiangiogenic action against the highly vascularized CT-2A mouse astrocytoma, which primarily expresses complex gangliosides. Brain tumors expressing high levels of GM3 are generally less vascularized and grow slower than tumors that express low levels of GM3. GM3 inhibits angiogenesis through autocrine and paracrine effects on vascular endothelial growth factor (VEGF) and associated receptors. GM3 should be a clinically useful compound for managing brain tumor angiogenesis.

## 1. Introduction

Malignant brain cancer persists as a catastrophic illness and is the second leading cause of cancer death in children [1–4]. The failure to effectively manage malignant brain cancer has been due in large part to the highly invasive nature of the disease and to the unique anatomical and metabolic environment of the brain, which prevents the large-scale resection of tumor tissue and impedes the delivery of therapeutic drugs. Invasion/metastasis involves the dissemination of tumor cells from the primary neoplasm to surrounding tissue and distant regions. In addition, the invasive cells establish a microenvironment facilitating colonization (angiogenesis and further proliferation), resulting in macroscopic malignant secondary tumors [5, 6]. Tumor cell invasion is correlated with tumor angiogenesis (vascularity), as prognosis is generally worse for brain tumors that are more vascular than for those that are less vascular [7–9]. Consequently, therapies that can simultaneously target both angiogenesis and invasion could provide effective longer-term management of malignant brain cancer.

## 2. Glycosphingolipids and Angiogenesis

**Figure 1:**
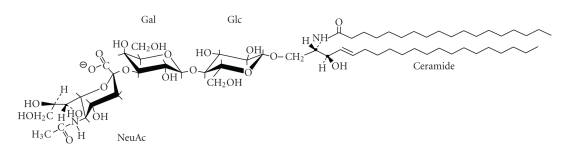
Structure of Ganglioside GM3 (NeuAc-alpha 2→ 3Gal-beta1 → 4Glc-beta1 →1′ Ceramide) (from [14] with permission).

Gangliosides are a family of cell surface-enriched glycosphingolipids that have long been implicated in tumorigenesis [10–12]. These molecules contain an oligosaccharide head group attached to a lipophilic ceramide, consisting of a sphingosine base and a long-chain fatty acid (Figure 1). The presence of sialic acid (N-acetylneuraminic acid, NeuAc) distinguishes the gangliosides from other glycosphingolipids. Gangliosides are anchored in the outer surface of plasma membranes through their ceramide moiety, which allows the head group to modulate numerous cell surface events such as growth, migration, adhesion, and signaling [12–15]. 

The structurally simple monosialoganglioside GM3 contains a single terminal sialic acid (Figure 1). N-acetylneuraminic acid is the predominant sialic acid species expressed in mammalian brain gangliosides [16, 17]. In contrast to N-acetylneuraminic acid, N-glycolylneuraminic acid is a predominant sialic acid species expressed in gangliosides from nonneural tissues of most nonhuman species (rodents, bovine, etc.) [17]. As humans lack the gene for the synthesis of N-glycolylneuraminic acid [18, 19], expression of N-glycolylneuraminic acid in gangliosides of human cells or tissues is attributed to contamination from exposure to nonhuman serum or from diet [17, 20, 21]. The involvement of gangliosides in angiogenesis is dependent on the intact molecules as neither asialo species nor sialic alone influence angiogenesis [22]. 

GM3 modulates the function of several receptors implicated with angiogenesis to include those for the insulin-like growth factor-1 (IGF-1), basic fibroblast growth factor (b-FGF), epidermal growth factor (EGF), platelet-derived growth factor (PDGF), vascular endothelial growth factor (VEGF), and cell adhesion molecules including the integrins [7, 12, 23–28]. GM3 also reduces proliferation and enhances apoptosis of rapidly proliferating neural stem cells [29]. Furthermore, Alessandri, Ziche, and coworkers originally found that several complex gangliosides (GM2, GM1, GD3, GD1a, GD1b, and GT1b) enhanced the action of angiogenic inducers, whereas ganglioside GM3 was inhibitory [30–32]. These observations suggest that GM3 could have therapeutic potential against tumor cell proliferation and angiogenesis.

The ratio of GM3 to the proangiogenic gangliosides GD3 and GD1a (GM3/GD3; GM3/GD1a) is lower in more metastatic and aggressive tumors than that in less metastatic tumors [7, 33–35], suggesting that elevated expression of complex gangliosides enhances tumor malignancy. In contrast to most human glioma tumor tissues, which contain high levels of the pro-angiogenic ganglioside GD3 [36–41], GD3 is not heavily expressed in mouse brain tumors or in most cultured human brain tumor cells [17, 42–44]. Although GM3 is also expressed in malignant human brain tumors, we think that GM3 expression in these tumors might serve to regulate or to counteract the pro-angiogenic action of GD3 and other complex gangliosides.

## 3. Evidence Supporting the Anti-Angiogenic Action of GM3 in Brain Cancer

Table 1 summarizes data from our previous studies on the association of GM3 expression with the angiogenic properties of multiple experimental mouse and human brain tumor models [17, 44, 45]. This survey shows that brain tumors with high GM3 expression are less angiogenic (vascularized) than brain tumors with low GM3 expression. GM3 expression was also correlated with greater cell-cell adhesion and slower growth [14, 44]. We later showed that the gene-linked knockdown of GM3 expression in the experimental ependymoblastoma (EPEN) tumor, which contains GM3 as the only ganglioside, increased vascularity (angiogenesis) [34]. An opposite effect was observed in the highly angiogenic CT-2A astrocytoma when we upregulated GM3 expression (below). These and other findings led us to conclude that the ratio of GM3 to complex gangliosides (GM1, GD1a, GT1b) can influence the angiogenic properties of a broad range of brain tumor types and are consistent with previous findings on the role of GM3 in other systems [22, 29, 32, 41, 46–49].

## 4. Anti-Angiogenic Action of GM3 in the CT-2A Astrocytoma

**Figure 2:**
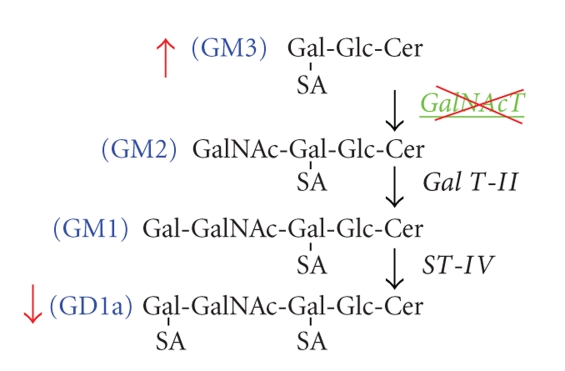
Pathway for the synthesis of ganglioside GM2 from GM3 by Gal*N*Ac-T. Gal*N*Ac-T adds a beta-linked *N*-acetylgalactosamine residue to the galactose of GM3 to form GM2, a key step required for the synthesis of complex gangliosides, GM2, GM1, and GD1a. Antisense targeting of the *GalNAc-T* gene reduces GD1a content, while increasing GM3 content [7].

**Figure 3:**
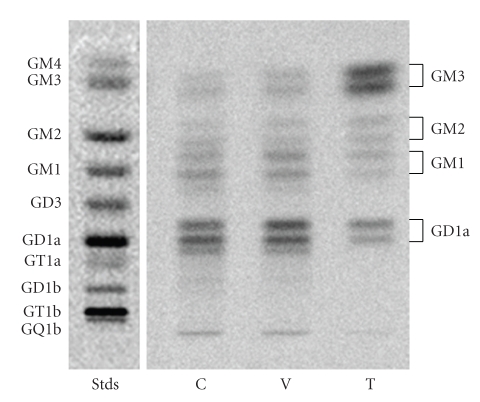
High-performance thin-layer chromatographic analysis of ganglioside distribution in the CT-2A astrocytoma: Control untransfected CT-2A (C), CT-2A transfected with empty vector alone (V), and CT-2A transfected with the antisense sequence to the *GalNAc-T* gene (T). Synthesized gangliosides appear as double bands due to ceramide structural heterogeneity. Analysis of synthesized gangliosides and standards (left lane) was as we described [7]. Knockdown of the *GalNAc-T* gene elevated GM3 content, while reducing GD1a content in the antisense T cells.

The CT-2A astrocytoma was produced following implantation of the chemical carcinogen, 20-methylcholanthrene, into the cerebral cortex of C57BL/6J mouse according to the procedures of Zimmerman and Arnold [44, 50]. The CT-2A tumor grows rapidly, is deficient in the phosphatase and tensin homologue/tuberous sclerosis complex 2, and is highly angiogenic [7, 51, 52]. We used an antisense construct to inhibit GalNAc-T expression in CT-2A cells as shown in (Figure 2). This caused a significant shift in ganglioside distribution, elevating GM3 content while reducing GD1a content (Figure 3).

**Figure 4:**
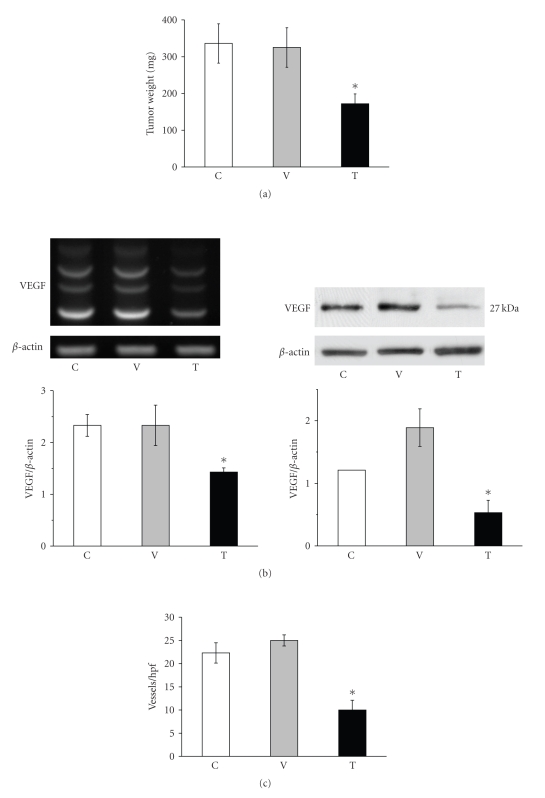
Ganglioside shift reduces growth, VEGF gene and protein expression, and vascularity in the CT-2A astrocytoma: Control untransfected CT-2A (C), CT-2A transfected with empty vector alone (V), and CT-2A transfected with the antisense sequence to the *GalNAc-T* gene (T). (a) The values are expressed as mean mg wet weight ± SE.*: significant compared to control C and V tumors at the *P* < .01 level. CT-2A (*n* = 10), CT-2A/V (*n* = 12), and CT-2A/TNG (*n* = 14) independent tumors. (b) RT-PCR and Western blot for VEGF. Other details are in [7]. (c) Vascularity determined using factor VIII-immunostained microvessels per ×200 field (hpf, high-powered field) from tissue sections as we described [7].

**Figure 5:**
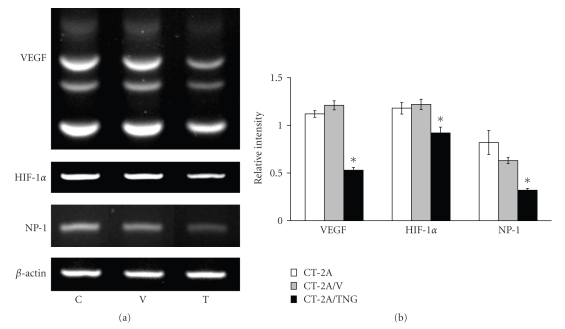
Ganglioside shift reduces VEGF, HIF-1*α*, and NP-1 gene expression in CT-2A- cultured cells: Control untransfected CT-2A (C), CT-2A transfected with empty vector alone (V), and CT-2A transfected with the anti-sense sequence to (a) the *GalNAc-T* gene (T). VEGF (multiple splice variants: 400–600 bp), HIF-1*α* (365 bp), and NP-1 (551 bp) amplification products were detected in each tumor cell line. Experimental conditions are as we described [7]. The gene to *β*-actin levels are expressed as the means of three independent samples ± SE.*: Significant compared to control C and V cells at (b) the *P* < .01.

The shift in ganglioside distribution significantly reduced growth, VEGF gene and protein expression, and blood vessel density in the orthotopically grown CT-2A tumors (Figure 4). Moreover, the shift in ganglioside distribution reduced gene expression for hypoxia inducible factor 1a (HIF-1*α*) and the VEGF coreceptor neruropilin-1 (NP-1) in the CT-2A cultured cells (Figure 5). This is interesting as HIF-1*α* is a transcription factor that regulates VEGF expression through the PI-3k/Akt signaling pathway [51, 53–55]. Viewed collectively, these data show that endogenous upregulation of GM3 reduces growth and angiogenesis in the rapidly growing and highly vascularized CT-2A mouse astrocytoma.

**Figure 6:**
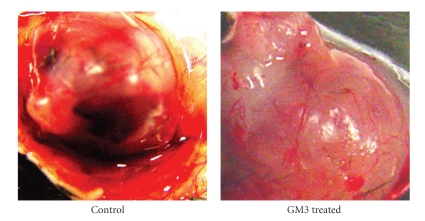
GM3 reduces CT-2A tumor vascularity when added to the tumor microenvironment. Small fragments of the CT-2A tumor were grown in Matrigel that contained either no GM3 (control) or GM3 (40 *μ*M). The tumor was grown in Matrigel for approximately two weeks in the flank of the syngeneic host C57BL/6 mice according to our standard procedures [7, 34]. Florid vascularization and the number and size thrombotic vessels were noticeably less in the presence than in the absence of GM3. Similar results were found in two independent experiments.

It was initially unclear, however, whether it was the elevation of GM3, the reduction of the pro-angiogenic ganglioside GD1a, or the change in GM3/GD1a ratio that was responsible for the reduction in CT-2A angiogenesis. It is well documented that gangliosides are shed from tumor cells into the microenvironment where stromal (endothelial) cells take them up to influence tumor progression [56–60]. Our most recent findings show that GM3, by itself, markedly reduces CT-2A vascularity when grown in the in vivo Matrigel model (Figure 6). These findings suggest that GM3 could be applied as a drug therapy directly to the tumor site and to surrounding areas following surgical tumor resection in humans. Alternatively, GM3 could be applied in liposomes as a pharmacotherapy for preformed tumors. Our findings in brain tumor cells are also consistent with previous findings in rabbit cornea showing that GM3 applied directly to tissue is anti-angiogenic [32]. Viewed, collectively, our findings indicate that GM3 has powerful anti-angiogenic action against the CT-2A astrocytoma when present in the microenvironment and can counteract the pro-angiogenic effects of complex gangliosides.

**Figure 7:**
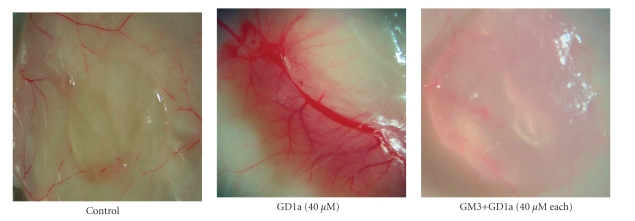
GM3 inhibits the pro-angiogenic effects of GD1a in the in vivo Matrigel assay. Matrigel alone (control) or containing GD1a or GD1a with GM3 was injected subcutaneously (s.c.) in SCID mice as we described [49]. Plugs were photographed (12.5×) on day 7 after Matrigel injection.

**Figure 8:**
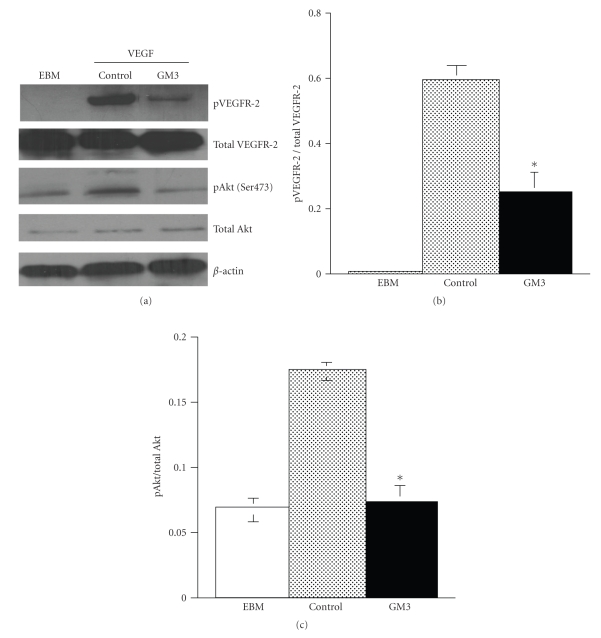
GM3 inhibits VEGFR-2 and Akt phosphorylation in HUVEC. HUVECs were incubated with GM3 (100 ng/ml) in endothelial basal medium (EBM) for 24 hours and were then stimulated with VEGF (100 ng/ml) for 5 minutes as we described [49]. (a) Cell lysates were prepared and measurement of VEGFR-2 and Akt phosphorylation over total was analyzed using Western blots [49]. (b) VEGFR-2 and (c) Akt phosphorylation were significantly lower in GM3-treated HUVEC than in control HUVEC (*P* < .001). Values are expressed as means ± SEM (*n* = 3 independent experiments).

Further evidence for a direct anti-angiogenic role of GM3 came from our recent studies with human umbilical vein endothelial cells, HUVEC. We found that GM3, by itself, significantly suppresses VEGF-induced proliferation and migration of HUVEC [49]. Moreover, GM3 significantly blocks GD1a-induced angiogenesis in the in vivo Matrigel assay (Figure 7). GD1a is a complex ganglioside associated with enhanced angiogenesis [7, 61]. The suppression of VEGF receptor 2 and Akt phosphorylation underlies the anti-angiogenic effect of GM3 on HUVEC (Figure 8). Additionally, the EPEN tumor, which expresses only GM3, has few blood vessels relative to tumors that express complex gangliosides [44, 45]. Consistent with our findings, Chung and coworkers recently showed that GM3 could suppress angiogenesis through the inactivation of VEGF-induced signaling by direct interaction with VEGFR-2 [47]. GM3 treatment could also reduce in vivo vascularity in the Lewis lung carcinoma model [47], while van Cruijsen et al. showed that vascularity was less and patient survival was better for nonsmall cell lung carcinomas that contained more GM3 than less GM3 [62]. Hence, GM3 is anti-angiogenic through its inhibition of the proangiogenic actions of complex gangliosides as well as through its direct inhibition of endothelial cell growth.

In summary, our results show that GM3 inhibits brain tumor angiogenesis. GM3 targets both tumor cells and endothelial cells. Although GM3 is elevated in human malignant brain tumors, its concentration is less than that of complex gangliosides especially GD3. We suggest that increasing the ratio of GM3 to complex gangliosides may be effective in reducing angiogenesis and growth in human glioblastomas. Our findings suggest that pharmacological application of GM3 is warranted as a potential nontoxic anti-angiogenic therapy for malignant brain cancer.

## Figures and Tables

**Table 1:** Association of GM3 levels to the vascularity of experimental brain *t* *u* *m* *o* *r* s*.

	Ganglioside Distribution		Vascularity
Brain Tumors	GM3	Complex Gangliosides	
Mouse			
EPEN	High	Low	Low
CBT-1	High	Low	Low
CBT-3	High	Low	Low

CBT-4	Low	high	High
CT-2	Low	High	High
CT-2A	Low	High	High

Human			
U87MG	Low	High	High

*All mouse brain tumors were produced from implantation of 20-methylcholantherene into the ventricle (EPEN), the cerebrum (CT), or the cerebellum (CBT) of C57BL/6J mice as we previously described [43].
